# Is an Idea Different from Cake: Can You Have It and Eat It, Too? A Violation of Permanence in Information Consumption

**DOI:** 10.1371/journal.pone.0041490

**Published:** 2012-07-25

**Authors:** Shu Li, Yue-Ran Li, Yin Su, Li-Lin Rao

**Affiliations:** Institute of Psychology, Chinese Academy of Sciences, Beijing, China; University of Minnesota, United States of America

## Abstract

One of the basic features of information is permanence. This feature states that the consumption of information by one consumer does not affect the availability of that information to other consumers. We present examples in two studies indicating that both laymen and experts of information exchange may be motivated to make choices that violate the permanence feature in *accepting* (Study 1) and *offering* (Study 2) information. When they possess, but have not yet consumed information, people may suffer from the appearance of wastefulness. This apparent wastefulness may provide a potential explanation for the observed violation of the permanence feature. Our findings indicate that, as the material age evolves into the information age, information-exchange behavior has not evolved significantly away from material-exchange behavior.

## Introduction

*“If you have an apple and I have an apple, and we exchange apples, we both still only have one apple. But if you have an idea and I have an idea, and we exchange ideas, we each now have two ideas.”*

*––George Bernard Shaw (1856–1950)*

We will begin with a personal experience of the author of this manuscript (Shu Li) that made him aware of the concept of information permanence many years ago. When a group of Final-Year-Project students were traveling on Singapore’s MRT (Mass Rapid Transit), a student, Ivy, offered to lend her friend a recently published fashion magazine to kill time but was rejected with a statement to the effect that the potential recipient had subscribed to the magazine and the issue would arrive that same day. Ivy later insisted that something must be wrong - she thought refusing to eat offered *dosa* or *lassis* (foods) would have been reasonable but refusing to read a magazine offered to occupy time while on a train was irrational. It is not surprising that Ivy was puzzled. A retrospective evaluation of such a decision reveals that the rejection of Ivy’s offer runs counter to the most distinguishing and frequently referred to feature of information consumption: that information is essentially permanent.

### The Permanence Feature

Ivy’s intuition that her friend’s refusal was irrational coincides with the permanence feature in information consumption. This concept states that the consumption of information by one consumer does not affect the availability of that information for use by other potential users [1]. Freiden et al. [2] developed a framework showing how information differs from both goods and services. They identified six characteristics that separate information from goods and services: heterogeneity, perishability, inseparability, tangibility, ownership, reproducibility. According to their definition, information differs from both goods and services in that information is not perishable [2]. Although some types of information, such as news, can become dated, information itself remains the same for all consumers [3]. Information may be uniformly consumed, and its consumption is non-rivalrous [1], [4]. One person’s consumption of a particular piece of information does not restrict others from also consuming it. The notion that consuming information does not use it up or change it constitutes a distinguishing feature of information that has been widely accepted without serious questioning [1], [2], [5], [6]. However, Ivy’s friend behaved in contrast to the permanence feature when she refused to take advantage of the free opportunity to read the magazine (information) as though she did not want to consume a concrete good (*e.g*., food and drink).

### The Psychology of Waste

What is likely to be responsible for the observed violation of the permanence feature in information consumption? The words of Ivy’s friend might give some clue as to the reason for this violation. The statement, “I have subscribed to this magazine, and this issue will arrive today *lah,*” implies an “as if” explanation, *i.e.*, “if I read the offered magazine to kill time, what will I do with the magazine I subscribed to?” In explaining why people make choices that compromise their own self-interest, Arkes (1996) suggested that people may eschew a behavior that is in their own best interest to avoid the appearance of wastefulness [7], [8]. We posited that a psychology of waste might also provide a potential explanation for violations of the permanence feature in information consumption.

### The Current Research

The goal of this study was to investigate whether the permanence feature would be violated in a situation similar to the one demonstrated by Ivy and her friend.

## Study 1a: Do we Accept Offered Information Differently from the Way we Accept Offered Material?

Why should we treat ideas differently from cake? Presumably we should do so because we cannot eat a piece of cake and have it too, but we can share an idea and still have it. The first experiment in Study 1 was designed to test whether people would accept offered information differently from an offered material good, as predicted by the permanence feature.

### Methods

Two hundred and nineteen graduate students (127 males, 92 females) at the Graduate University of the Chinese Academy of Sciences were recruited to fill out a short questionnaire in exchange for a small gift. A two-factor within-subject design was employed with the factors being ***goods-type*** (material, information) and ***goods-possession*** (possessed, not possessed). The questionnaires presented to the participants were composed of four hypothetical scenarios, which were presented with the order balanced across the subjects. The scenarios, which we call the *Accepting Problem,* read as follows, with the only differences between the scenarios in parentheses:

*Suppose you were going on a group tour on which you did not know any of the others in the group. On the trip, one group member spontaneously offered (* ***you a chance to view a DVD of a movie you have never viewed/you a roll of film you have not brought*** *) valued at ¥150* ***.*** *Would you accept the offer if you* ***(had/had never)*** *purchased one?*

Following each scenario, participants were instructed to rate their willingness to accept or reject the stranger’s offer on a 4-point scale from 1 (definitely willing to accept) to 4 (definitely willing to reject). The study was approved by the Institutional Review Board of the Institute of Psychology, the Chinese Academy of Sciences. Because the protocol was judged to pose low risk and the data were analyzed anonymously, oral consent was recommended and obtained from study participants before data collection.

### Results and Discussion

**Figure 1:**
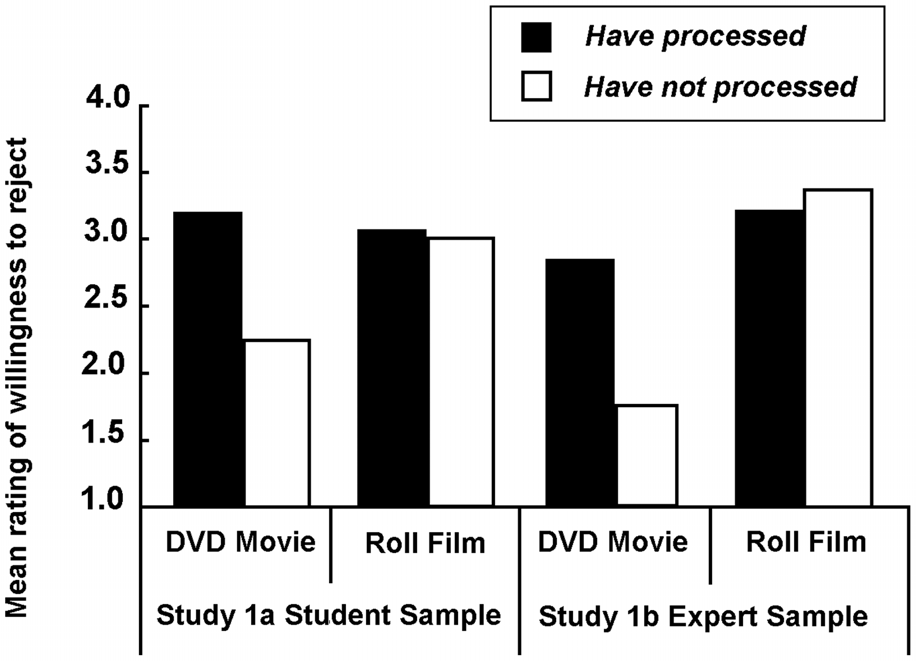
Mean ratings of willingness to accept or reject the stranger’s offer in Study 1 (1 definitely willing to accept, 4 definitely willing to reject).

The results are summarized in Figure 1. A two-factor, repeated-measures ANOVA of participants’ rating of willingness to accept or reject the stranger’s offer revealed a significant goods-type effect (*F*(1,218) = 40.823, *p*<.001), a significant goods-possession effect (*F*(1,218) = 81.628, *p*<.001) and a significant interaction effect, *F*(1,218) = 79.970, *p*<.001. A simple effect analysis of the interaction showed that people were consistently willing to reject the offered roll of film regardless of whether they had possessed it (*M* = 3.12) or not (*M* = 3.10), *F*(1,218) = .116, *n.s*. In contrast, people were more willing to accept the offered DVD they had never possessed (*M* = 2.22) but prone to reject if they already had it (*M* = 3.18), *F*(1, 218) = 132.804, *p*<.001.

The results of Study 1a showed that people treated information and material items offered by others differently. When faced with a material offering, the participants were equally reluctant to accept the material regardless of whether they had possessed the item or not. The film used in our questionnaire was treated exactly the same as *a piece of cake,* which has been aptly described as something that you cannot have and eat, too. When faced with an offer of information, however, people were more reluctant to accept the information if they already possessed it, but had not yet consumed it than if they did not possess it. The DVD used in our questionnaire was treated exactly the same as an *idea* (which we can share and still have, as characterized by the permanence feature) when our participants had not possessed it but somewhat like a piece of *cake* when our participants had possessed but not yet consumed it. The latter case constitutes an “Ivy-type” violation of the permanence feature in the sense that the availability of a piece of information was affected by the consumption of that information.

## Study 1b: Do Experts of Information Exchange behave Differently?

Experts have been shown to make more accurate and reliable judgments than novices in a variety of fields [9], suggesting that experts of information exchange might be more likely to understand the permanence feature. In addition, because what was experienced by Ivy’s friend is similar to experiencing a sense of wanting to avoid appearing wasteful, we reasoned that the “psychology of waste” proposed by Arkes [7] could constitute a possible explanation.

Study 1b was therefore designed with a two-fold goal. The first was to examine whether the violation of the permanence feature would be absent from an expert sample. The second was to examine whether the observed violation could be explained by the “psychology of waste” [7].

### Methods

Following the same procedure used in Study 1a, data were collected from one hundred full-time employees (50 males, 50 females) of the State Intellectual Property Office of the People’s Republic of China (SIPO), who were well-trained in the field of information exchange and thus presumably more sensitive to the permanence feature. In order to test the “Psychology of Waste” explanation, all respondents were asked to indicate which of the following best described their rationale for accepting or rejecting the stranger’s offer:

- *The offered product could be re-used by the provider if I used it.*
- *The offered product could not be re-used by the provider if I used it.*
- *It would be a waste of my previously purchased product.*
- *Other (Please write down your reason in the space provided)*

The study was approved by the Institutional Review Board of the Institute of Psychology, the Chinese Academy of Sciences. Because the protocol was judged to pose low risk and the data were analyzed anonymously, oral consent was recommended and obtained from study participants before data collection.

### Results and Discussion

The mean rating of the willingness to accept or reject the stranger’s offer is shown in Figure 1. The two-factor, repeated-measures ANOVA of the participants’ rating of willingness to accept or reject the stranger’s offer closely corresponded to the ANOVA from Study 1a. That is, there was a significant goods-type effect (*F*(1,99) = 143.072, *p*<.001), a significant goods-possession effect (*F*(1,99) = 30.675, *p*<.001) and a significant interaction effect, *F*(1,218) = 50.911, *p*<.001.

Combining the data from the expert sample with the data from the student sample, a three-factor (goods-type, goods-possession and sample-type) analysis revealed that no significant difference existed between the student sample and the expert sample (*F*(1,317) = 2.458, *n.s.*) and that no significant interaction occurred between the three factors (*F*(1,317) = 1.561, *n.s.*). Apparently the permanence feature in information consumption was not considered even when the scenarios were posed to professionals who worked at the State Intellectual Property Office (SIPO).

**Table 1:** The percentage of expert participants endorsing each option for the various scenarios (%, n = 100).

Goods-type (Information, Material)	Information		Material	
Goods-possession (Yes, No)	Yes	No	Yes	No
(a) The offered product could be re-used by the provider if I used it.	28	86	0	0
(b) The offered product could not be re-used by the provider if I used it.	0	8	72	72
(c) It would be a waste of my previously purchased product.	66	0	0	4
(d) Other.	6	6	28	24

Reasons for accepting or rejecting the stranger’s offer are presented in Table 1. All four options received some endorsement. Roughly three-quarters of our expert participants considered that “the offered product could not be re-used by the provider if I used it” in each of the two material-good scenarios. This choice distribution may explain why people consistently rejected the material goods. In contrast, when they were offered a DVD that they had not purchased, more than eighty percent of the responders thought that “the offered product could be re-used by the provider if I used it”. The concept of reusability may explain why people were willing to accept an offered DVD that they had not possessed. Accepting a piece of cake will inevitably lead to a loss to the provider; whereas accepting an idea will not. Therefore, it made sense that they rejected the offered material but accepted the offered information.

However, when they were offered a DVD that they had previously purchased, a majority of the experts of information exchange (more than sixty percent) would no longer take into account that “the offered product could be re-used by the provider if I used it”, but instead thought that it was “a waste of my previously purchased product”. The emergence of waste as a consideration may provide a possible explanation as to why people were prone to reject the information-goods they already possessed.

The result of Studies 1a and 1b consistently showed that the permanence feature in information consumption was obeyed when participants did not already possess the offered information but was violated when participants already possessed the offered information. The “psychology of waste” appears to explain this observed violation of the permanence feature.

## Study 1c: Would you Reject Reading a Magazine for Free?

In Study 1c, we went one step further in an attempt to determine whether people made choices that were contrary to the permanence feature through real-life responses rather than through responses to hypothetical scenarios. The rationale behind this experiment was to test the idea that if there was motivation to avoid the appearance of wastefulness, people might be reluctant to utilize an identical piece of information that was available before utilizing a piece of information they already possessed.

### Methods

Forty-three graduate students (26 males, 17 females) at the Graduate University of the Chinese Academy of Sciences participated in this experiment.

The participants were recruited to participate in an unrelated experiment on brand recognition. Before participating in the brand experiment, they were led to separate breakout rooms upon arrival and told that they would be given a magazine for participating in the brand experiment. Participants were offered two back issues of a very popular Chinese magazine (*Reader*) and were asked to choose one to be mailed to their postal address. The selected issue was then put into an envelope and addressed by the participant.

While they were waiting for the brand experiment, participants were asked whether they would like to read an identical copy of the selected magazine to kill time. If they rejected the offer, they were then asked whether they would like to read an identical copy of the magazine they had not selected to kill time.

The participants’ decisions about whether to read or not, as well as their reports of whether they had read the two magazines before, were recorded. The envelopes with the selected magazines were sent to the participants after the experiment. The study was approved by the Institutional Review Board of the Institute of Psychology, the Chinese Academy of Sciences. Because the protocol was judged to pose low risk and the data were analyzed anonymously, oral consent was recommended and obtained from study participants before data collection.

### Results and Discussion

**Figure 2:**
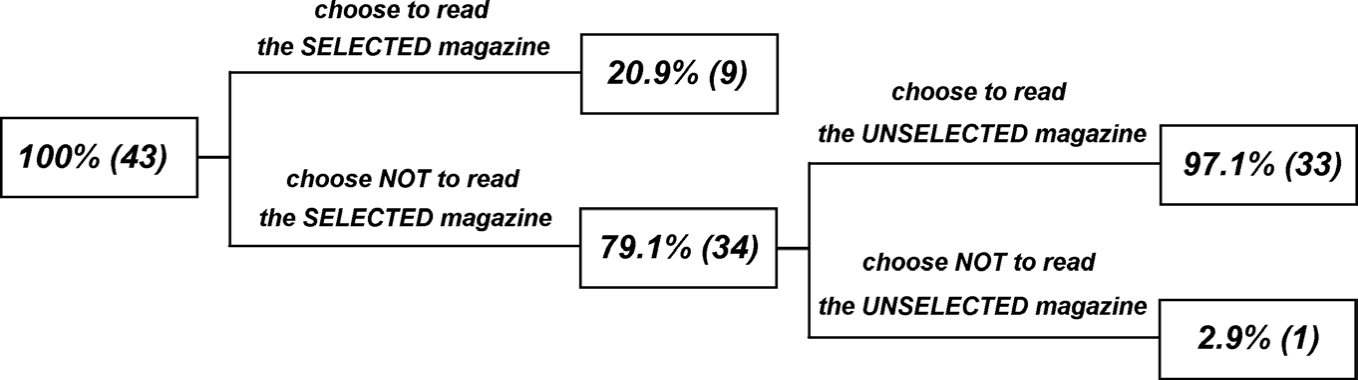
A decision tree employed to characterize reading options.

A decision tree was used to characterize reading options and outcomes (see Figure 2). All participants reported that they had not read either of the two issues before.

Of the 43 participants observed, 79.1 percent (34) chose NOT to read the magazine that would be sent to them, while 20.9 percent (9) chose to read the magazine that would be sent to them. Of the 34 participants who chose not to read the chosen magazine, 97.1 percent (33) chose to read the magazine that would NOT be sent to them, while 2.9 percent (only 1 person) consistently chose not to read either magazine. A Chi-square test showed that participants were more reluctant to read the magazine that they selected to be sent to them (*p*<.001). However, the test showed that they were overwhelmingly willing to kill time by reading the magazine that would NOT be sent to them (*p*<.001). This latter finding indicates that the participants were motivated to kill time by reading a free magazine.

The participants’ actual responses (*i.e*., the majority of the participants were reluctant to read the magazine that would be sent but were willing to read the magazine that would not be sent to themselves) strengthens the idea that the permanence feature is considered in the absence of psychological waste but violated in the presence of it.

Summarizing Study 1, both the hypothetical and actual responses of our participants indicated that people would not accept a piece of offered information if they already possessed it, violating the permanence feature. Arkes’s “Psychology of Waste” seems to explain our findings. As information consumption consists of both the receiving and the offering of information, Study 2 was conducted to test the robustness of the detected violation from the perspective of information offering.

**Figure 3:**
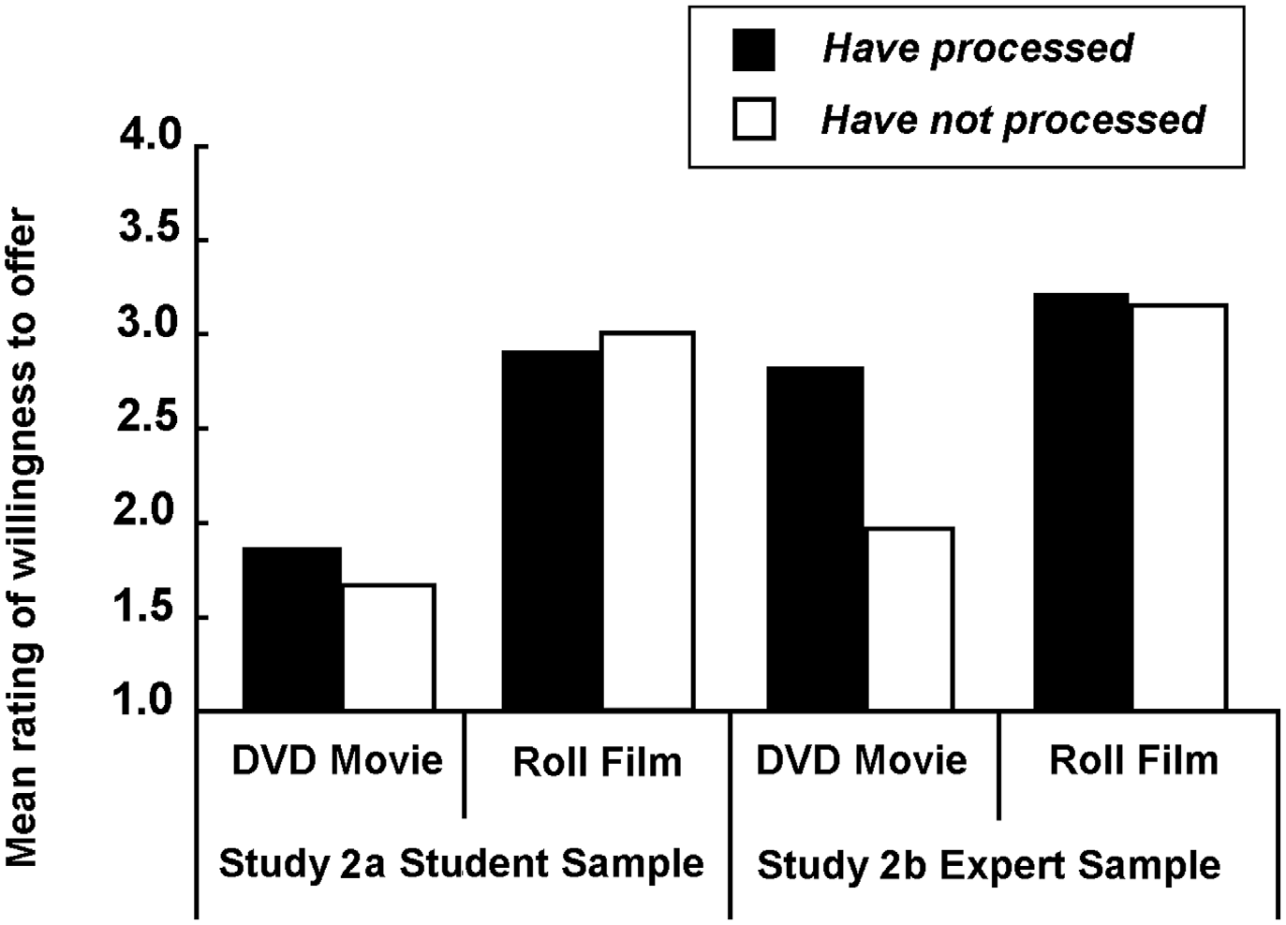
Mean ratings of willingness to provide a stranger with an offer in Study 2 (1 definitely willing to offer, 4 definitely unwilling to offer).

## Study 2a: Are we as Unwilling to Offer Information to Others as we are to Offer Material?

Study 1 addressed the question of whether information-receivers would utilize a source of information if they perceived that the information would be wasted. Study 2, on the other hand, sought to answer the question of whether information-givers would offer information to others if they perceived that the recipients would waste the information. If the results are also negative, the violation would appear to be even more counterintuitive because the potential wastefulness is not that of the person making the offer but the recipient’s.

### Methods

The design of Study 2a was similar to that of Study 1a. One hundred and twenty graduate students (68 males, 52 females) at the Graduate University of the Chinese Academy of Sciences were recruited to fill out a short questionnaire in exchange for a small gift. The scenarios, which we call the *Offering Problem,* were modified from the Accepting Problem in Study 1a and read as follows, with the only differences between the scenarios in parentheses:

*Suppose you were going on a group tour on which you did not know any of the others in the group. You carried with you* ***(a DVD of a movie/a roll of film)*** *valued at ¥150. On the trip, you found one group member that* ***had never viewed the movie/had not brought the same kind of film*** *. Would you offer the group member* ***(the DVD/the film)*** *if he/she* ***(had previously/had never)*** *purchased one?*

Following each scenario, the participants were instructed to rate their willingness to offer the stranger the DVD/film on a 4-point scale from 1 (definitely willing to offer) to 4 (definitely unwilling to offer). The study was approved by the Institutional Review Board of the Institute of Psychology, the Chinese Academy of Sciences. Because the protocol was judged to pose low risk and the data were analyzed anonymously, oral consent was recommended and obtained from study participants before data collection.

### Results and Discussion

Figure 3 summarizes the results from Study 2a. A two-factor, repeated-measures ANOVA revealed no goods-possession effect (*F*(1,119) = 2.412, *n.s.*) on the rating of willingness to provide a stranger with an offer, but a significant goods-type effect (*F*(1,119) = 138.103, *p*<.001), and a significant interaction effect, *F*(1,119) = 10.476, *p*<.001. A simple effect analysis of the interaction showed that people were consistently unwilling to offer a roll of film regardless of whether the stranger already possessed one (*M* = 2.89) or not (*M* = 2.96) (*F*(1,119) = 1.067, *n.s.*). In contrast, people were more willing to offer a DVD that the stranger did not possess (*M* = 1.63), but less willing to offer one if the stranger already possessed it (*M* = 1.88), (*F*(1, 119) = 8.688, *p*<.01). Therefore, a violation of the permanence feature was also observed from the perspective of offering information.

## Study 2b: Do Experts of Information Exchange Respond Differently from Non-experts when they Perceive that Others may Potentially Waste Information?

Following the same logic as Study 1b, an expert sample rather than a student sample was used to test whether experts of information exchange are more immune to the violation.

**Table 2:** The percentage of participants who endorsed each option in the various scenarios (%, n = 123).

Goods-type (Information, Material)	Information		Material	
Goods-possession (Yes, No)	Yes	No	Yes	No
(a) The offered product could be re-used by me if the recipient used it.	26.8	72.4	0.8	1.6
(b) The offered product could not be re-used by me if the recipient used it.	0.8	0	81.3	82.1
(c) It would be a waste of the recipient’s previously purchased product.	62.6	0	0.8	0.8
(d) Other.	9.8	27.6	17.1	15.4

### Methods

The material and procedure in Study 2b were exactly the same as Study 2a except that a test of the “Psychology of Waste” was added. Participants were asked to indicate which of the following best described their reasons for offering or not offering the DVD/film:

- *The offered product could be re-used by me if the recipient used it.*
- *The offered product could not be re-used by me if the recipient used it.*
- *It would be a waste of the recipient’s previously purchased product.*
- *Other (Please write down your reason in the space provided.).*

The data were collected from 123 full-time employees (64 males, 59 females) at SIPO. The study was approved by the Institutional Review Board of the Institute of Psychology, the Chinese Academy of Sciences. Because the protocol was judged to pose low risk and the data were analyzed anonymously, oral consent was recommended and obtained from study participants before data collection.

### Results and Discussion

A two-factor, repeated-measures ANOVA of the experts’ rating of willingness to offer information/material to a stranger (Figure 3) revealed a similar result to that of Study 2a except that the goods-possession effect turned out to be significant (*F*(1,122) = 30.347, *p*<.001).

Combining the data of the expert sample with that of the student sample, a three-factor (goods-type, goods-possession and sample-type) analysis revealed a significant difference between the student sample and the expert sample (*F*(1,241) = 33.002, *p*<.001), together with a significant interaction effect between the three factors (*F*(1,241) = 11.367, *p*<.05). Most interestingly, a simple effect analysis of the interaction showed that the experts of information exchange were more unwilling to offer information than were the students, especially in the “possessed but not consumed” condition (*F*(1,241) = 51.164, *p*<.001). This finding suggests that the permanence feature was violated more severely when the problems were posed to professionals who were assumed to have a clearer understanding of the feature.

The distribution of choice (see Table 2) was similar to that obtained in Study 1b. The fact that more than sixty percent of the responders thought “it would be a waste of the recipient’s previously purchased product” when they considered offering a DVD that the stranger had previously purchased, is noteworthy. The “Psychology of Waste” may again provide a possible explanation as to why people were unwilling to offer the information-goods to others.

The results show a more serious violation of the permanence feature: information*-givers* are less willing to offer information even when others are the ones who would waste the information.

## Study 2c: Would you Offer a Magazine if the Recipient will Eventually Own it?

Study 2c was an attempt to replicate the findings of Study 2a and 2b using actual behaviors rather than hypothetical scenarios. We inferred that people might be reluctant to offer a piece of information for free to someone who will eventually possess the same information.

**Table 3:** The percentage (number) of participants who offered a free read of the magazine in Study 2c.

	The issue to be mailed to the confederate researcher		
		Issue 8 (*n* = 21)	Issue 9 (*n* = 21)
The issue offered by the actual participant	Issue 8	23.8% (5)	71.4% (15)
	Issue 9	76.2% (16)	28.6% (6)

### Methods

Forty-two graduate students (25 males, 17 females) at the Graduate University of the Chinese Academy of Sciences participated in this experiment. The participants were recruited to participate in an unrelated experiment on decision making. In our experiment, one actual participant and one undercover researcher were seated in a classroom. The real participant was offered two back issues of the same popular Chinese magazine as was used in Study 1c for participating in the decision making experiment. Meanwhile, the researcher was offered one of the two back issues in the presence of the actual participant and asked to put it into a self-addressed envelope to be sent by mail.

While they were waiting for the upcoming decision making experiment, the actual participant was asked whether he/she would be willing to offer one of his/her two issues to the “bored” researcher to kill time. The participant’s decision about which issue to offer was recorded. The study was approved by the Institutional Review Board of the Institute of Psychology, the Chinese Academy of Sciences. Because the protocol was judged to pose low risk and the data were analyzed anonymously, oral consent was recommended and obtained from study participants before data collection.

### Results and Discussion

The participants’ decisions are summarized in Table 3. A chi-square test showed that the issue that the participant chose to offer to the undercover researcher was significantly related to the one selected to be mailed to the researcher (*p*<.05). Specifically, of the 21 participants in scenarios in which Issue 8 was to be mailed to the confederate researcher, 76.2 percent (16) chose to offer the other issue (Issue 9) to the “bored stranger”; whereas only 23.8 percent (5) chose to offer the same issue (Issue 8). Similarly, in the situation in which Issue 9 was to be mailed to the researcher, 71.4 percent (15) of the 21 participants chose to offer the other issue (Issue 8); whereas only 28.6 percent (6) chose to offer the same issue (Issue 9).

Study 2c provided behavioral evidence that considering the possible wastefulness of another may also lead to a violation of the permanence feature.

Taken together, both the hypothetical and real decisions in Study 2 generally demonstrated that the violation of the permanence feature from the perspective of information offering was robust.

## Study 3: Which is More Responsible for the Observed Violation, the Psychology of Waste or the Endowment Effect?

An earlier study found that the subjective value of information follows the predictions of endowment effect theory [10]. According to this theory, if mere ownership increases an individual’s valuations of objects, people might place more value on the magazines that they already possess than on newly offered ones, leading to the rejection of the newly offered magazine. Therefore, the endowment effect might be an alternative account for the observed violation of the permanence feature. We conducted Study 3 to test which explanation, the “psychology of waste” or the endowment effect, is more responsible for the observed violation.

**Figure 4:**
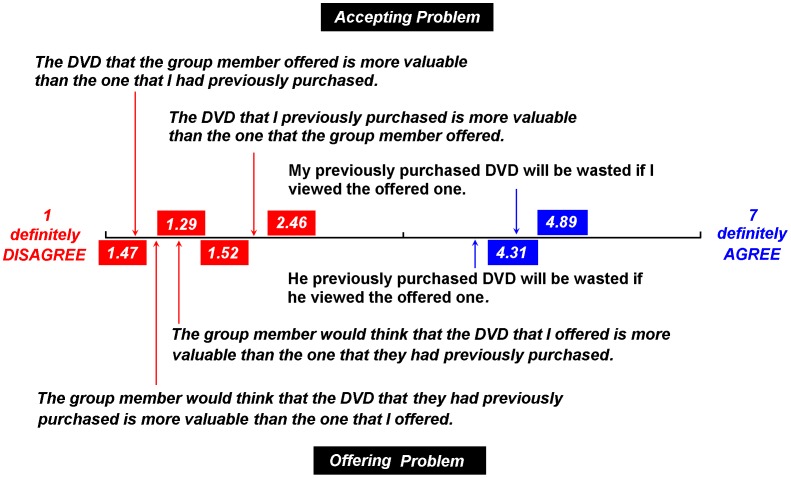
Mean ratings of agreement with each point supporting either the psychological waste account or the endowment account (1 definitely disagree, 7 definitely agree).

### Methods

Ninety-four full-time employees (47 males, 47 females) at SIPO were recruited to fill out a short questionnaire. Only the already possessed DVD condition (“*if you* ***have*** *previously purchased it” or “if he/she* ***had previously*** *purchased one*”) in both the Accepting Problem and the Offering Problem used in Studies 1 and 2 was presented to the participants. In order to counterbalance the order of scenario presentation, the problems were presented in two different versions (*i.e.*, the Accepting-Offering version and the Offering-Accepting version). Following each of the two possessed-information scenarios, the participants were asked to indicate their willingness to accept/offer the DVD on a 4-point scale.

Instead of presenting the questions previously used in Studies 1 and 2 to test the “psychology of waste” account, we asked the respondents to indicate the extent of their agreement with the following on a 7-point scale from 1 (definitely disagree) to 7 (definitely agree):

*“My/his previously purchased DVD will be wasted if I/he viewed the offered one.”*

In addition, to test for the endowment effect, we also asked the respondents to indicate the extent of their agreement with the following points on a 7-point scale from 1 (definitely disagree) to 7 (definitely agree):

***For the Accepting Problem***

*“The DVD that I previously purchased is more valuable than the one that the group member offered.”*

*“The DVD that the group member offered is more valuable than the one that I had previously purchased.”*

***For the Offering Problem***

*“The group member would think that the DVD that they had previously purchased is more valuable than the one that I offered.”*

*“The group member would think that the DVD that I offered is more valuable than the one that they had previously purchased.”*

A between-subject design was used in the present study. We randomly assigned the participants to one of the two versions. Of the 94 participants, 46 responded to the Accepting-Offering version and the remaining 48 responded to the Offering-Accepting version. The study was approved by the Institutional Review Board of the Institute of Psychology, the Chinese Academy of Sciences. Because the protocol was judged to pose low risk and the data were analyzed anonymously, oral consent was recommended and obtained from study participants before data collection.

**Figure 5:**
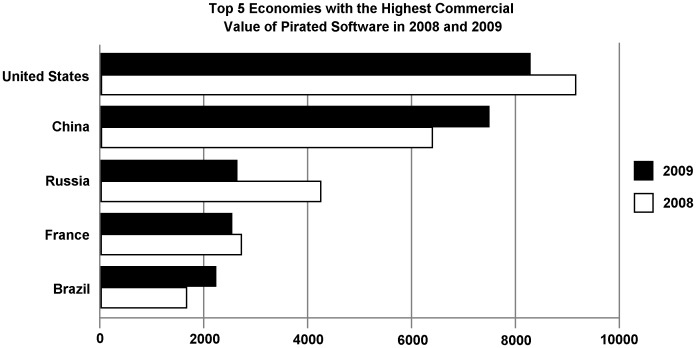
Commercial value of pirated software (in millions of US dollars) in the 5 economies with the highest commercial value in 2009. Data from the Business Software Alliance (BSA, 2010).

### Results and Discussion

Analyses of the mean rating of willingness (*M* = 3.32) to accept or reject the stranger’s DVD offer in the Accepting Problem and the mean rating of willingness (*M* = 2.94) to provide the stranger a DVD in the Offering Problem revealed similar results (*i.e.*, unwilling to accept/offer the DVD) to those obtained in Studies 1 and 2.

As shown in Figure 4, participants were prone to agree with the point *“My purchased DVD will be wasted if I viewed the offered one”* in the Accepting Problem, but disagreed with the point, “*The DVD that I previously purchased is more valuable than the one that the group member offered,”* and the point *“The DVD that the group member offered is more valuable than the one that I previously purchased.*” A similar result was obtained in the Offering Problem (see Figure 4). Study 3 supported the concept that the observed violation of the feature of the permanence could be explained by the “psychology of waste” but not by the endowment effect.

## General Discussion

During the long history of evolution, people have become accustomed to living in a world designed for material exchange. Although society has evolved into an information age, the characteristics associated with materials remain and are part of our daily lives. In this study, we found that the “psychology of waste” [7] can affect the availability of information. If someone knows that another possesses the same information, he or she may employ the behavior suggested by the “psychology of waste” and refrain from sharing. If a person knows that he/she is the only source for certain information, they may be more willing to share it. Such behavior violates the permanence feature suggested by information scientists *e.g.*, [1], [2], [5], [6] and indicates that as the material age evolves into the information age, information-exchange behavior has not evolved significantly away from material-exchange behavior.

“Waste not” is a rule that most of us have used in material-exchange behavior [8]. Yet to use this principle to reject available information represents an inappropriate application of this rule, which may be contrary to one’s own best interests. Furthermore, although information persists in that it can be used again and again, it can become dated (the feature of obsolescence) with newer information generally being more valuable than older information. The maladaptive tendency to apply the “waste not” rule to information usage is probably fostered by a colloquial notion of information, in which the value of information is unknowable [11]. In other words, information is viewed as an “experience good” in that its value is revealed only after use. If the participants had viewed the offered information in the scenarios used in this research study, the value of the possessed information would be lost or lessened. This concept is useful in measuring the value of information; however, in certain situations, it may also be maladaptive.

It should be noted that in the present study limit options were provided to explain the decisions for acceptance or offering. In Studies 1b and 2b, some participants selected the “other” option, indicating there were other reasons behind their decisions. As the aim of the present paper was to test whether the psychology of waste could provide a possible explanation, we did not further explore other reasons behind participants’ decisions. However, future research should explore other possibilities in greater detail.

Our findings, on one hand, imply that the impermanence feature of material consumption is deeply rooted in the human mind. On the other hand, it suggests that the permanence feature in information consumption is not as appealing as would be expected. In exploring the nature of material goods, we may eventually detect violations other than the “Ivy-type”. We believe that further interpretation of the results as well as further research is called for before making conclusions about the imperishable nature of information.

Every year, the governments of various countries, developed and developing alike, exert efforts to end piracy. For example, data from the *Business Software Alliance* shows that the worldwide commercial value of unlicensed software hit $51.4 billion and the piracy rate reached 43% in 2009 [12]. Figure 5 presents the top 5 economies ranked by the value of unlicensed software. A practical implication of our study is that perceived psychological waste may turn out to be a damping force on customers’ willingness to accept pirated products. For instance, if legitimate products could be placed in a low-priced pool for which people pay a flat entrance fee, using pirated products could be framed as ‘wasteful’. Accepting pirated products, therefore, could make all of the available legitimate products appear wasted. Our findings suggest that people would then be prone to reject the pirated products. A similar analysis applies to information-providers. Of course, such a pool would be an expensive system to set up, but making the use of legitimate products more feasible could be an important solution to the piracy problem. In addition, thinking about how much money has been poured into the war on piracy, directing a fairly large amount of money to facilitate the public’s perception of legitimate products appears an economical solution.
